# Efficiency Improvement Using Molybdenum Disulphide Interlayers in Single-Wall Carbon Nanotube/Silicon Solar Cells

**DOI:** 10.3390/ma11040639

**Published:** 2018-04-21

**Authors:** Shaykha Alzahly, LePing Yu, Cameron J. Shearer, Christopher T. Gibson, Joseph G. Shapter

**Affiliations:** 1 Flinders Centre for Nanoscale Science and Technology, College of Science and Engineering, Flinders University, Bedford Park, Adelaide, SA 5042, Australia; alza0112@uni.flinders.edu.au (S.A.); yu0252@uni.flinders.edu.au (L.Y.); cameron.shearer@flinders.edu.au (C.J.S.); christopher.gibson@flinders.edu.au (C.T.G.); 2 Department of Chemistry, The University of Adelaide, Adelaide, SA 5005, Australia; 3 Australian Institute for Bioengineering and Nanotechnology, University of Queensland, St. Lucia, QLD 4072, Australia

**Keywords:** molybdenum disulphide (MoS_2_), single-wall carbon nanotubes (SWCNTs), solar cells

## Abstract

Molybdenum disulphide (MoS_2_) is one of the most studied and widely applied nanomaterials from the layered transition-metal dichalcogenides (TMDs) semiconductor family. MoS_2_ has a large carrier diffusion length and a high carrier mobility. Combining a layered structure of single-wall carbon nanotube (SWCNT) and MoS_2_ with n-type silicon (n-Si) provided novel SWCNT/n-Si photovoltaic devices. The solar cell has a layered structure with Si covered first by a thin layer of MoS_2_ flakes and then a SWCNT film. The films were examined using scanning electron microscopy, atomic force microscopy and Raman spectroscopy. The MoS_2_ flake thickness ranged from 5 to 90 nm while the nanosheet’s lateral dimensions size ranged up to 1 μm^2^. This insertion of MoS_2_ improved the photoconversion efficiency (PCE) of the SWCNT/n-Si solar cells by approximately a factor of 2.

## 1. Introduction

Global energy demand has increased dramatically in recent years due to the rapid increase in world population, use of modern technologies and improved standards of living. According to the U.S Energy department, by 2040, the global energy demand will increase by 28% [1]. With the increasing global energy consumption and the commitment to reduce the amount of CO_2_ and other greenhouse gases emitted into the atmosphere due to the burning of fossil fuels, there is a great need to improve the current photovoltaic systems and develop other sources of renewable energy such as wind power [2], fuel cells [3], biofuels [4] and solar cell technologies [5]. These alternative energy technologies have the capability to meet the world’s energy demand if well developed. However, the challenge is that the production from these resources is small in relation to the required energy supply and are geographically limited to areas where the resource is in abundance and consistent. It is only solar energy that can be harnessed almost everywhere in the world, providing a possible solution to the current energy demand [6]. There are many solar photovoltaics of next generation including carbon nanotube-silicon (CNT/Si) heterojunction solar cells [7].

Since nanotube-silicon heterojunction solar cells were reported by Wei et al. in 2007, they have been widely studied because of their potential to replace the expensive p-type emitter layer in crystalline silicon solar panels that are in use today [8]. CNT/Si solar cells possess a high solar-to-electricity conversion efficiency and can be manufactured using simple, inexpensive materials using an easy fabrication procedure. Studies have reported 17% efficiency with the use of metal oxide layers for both efficient carrier transport and as an antireflection layer [9]. These findings present solid evidence that CNT/Si devices will possibly replace silicon solar cells [10]. Typically, the CNT/Si cell is similar to the conventional n-type silicon cell but in CNT/Si devices, a highly transparent film of CNT replaces the p-type silicon layer and the front metallization. In air, CNTs adsorb O_2_ and hence are p-type. When deposited on n-type Si, a depletion region is established and the built-in potential in this heterojunction can separate charge carriers to yield a current. CNT/Si cells are potentially cheap, semi-transparent, flexible, have excellent conductivity and efficient even under low light [11].

However, there are several important issues remaining that hinder the performance of CNT/Si-solar cells which need to be solved to allow commercial development. One issue that remains is the improvement of the Schottky barrier, which could be accomplished by using an effective carrier transfer layerto enable the transport of a high current without losses due to carrier recombination [9].

The CNT films typically used in CNT/Si solar cells consist of a mat of CNTs, in contact with one another, to form a conductive grid. The CNT film must be transparent so the film is very thin (<10 nm) or sparse. The resistance of thin films made with SWCNTs is largely due to the hopping of charge carriers from one tube to another. There is also a resistance transferring charge carriers across the CNT/Si interface. Two-dimensional (2D) materials like molybdenum disulphide (MoS_2_) can be successfully applied in this case because they will extend charge carrier lifetimes due to the long carrier diffusion length (200–500 cm^2^/Vs) [12] in the 2D material. Thus, MoS_2_ is capable of lowering the charge carrier resistance by both helping transfer across the interface and decreasing the number transfers between CNTs. This reduction in resistance means that the insertion of MoS_2_ could enhance efficiency [13].

MoS_2_ is made of stacked monolayers that are bonded together by weak van der Waals forces. The monolayers consist of S-Mo-S units that are hexagonally packed [14]. MoS_2_ is one of the most studied and widely applied nanoelectronic transition metal dichalcogenides (TMDs). Like other TMDs, the material in its bulk state is an indirect band gap semiconductor with a band gap of 1.2 eV [15,16]. When the material is formed into a monolayer, the band gap becomes a direct band gap of 1.9 eV [16]. Due to this effect, bulk MoS_2_ and its monolayer have been studied extensively [17], with considerable research conducted on the potential applications of monolayer MoS_2_ in 2D devices. The existence of the large band gap in the monolayer of MoS_2_ has found applications in areas such as field effect transistors with an on/off ratio as high as 10^8^ [18], integrated circuits [19], sensors [20] and logic operations [21].

There are two exfoliation methods to produce 2D MoS_2_: micromechanical exfoliation and liquid phase exfoliation [16]. Both methods begin with bulk MoS_2_ to produce flakes of MoS_2_. In micromechanical exfoliation, the flakes are produced by manual removal and transfer using adhesive tape. Liquid phase exfoliation involves mechanical means such as shearing, sonication, stirring, bubbling and grinding [22], or via atomic intercalation through solution chemistry [23].

MoS_2_ exhibits robust mechanical properties. It has good photo-responsivity when used as a monolayer, allowing use in innovative solar cell devices [24]. When MoS_2_ and CNTs are combined, they can provide novel photovoltaic devices with excellent performance. The performance of SWCNT/n-Si hybrid solar cells also depends on the thickness of the CNT film, particularly for films with an average transmittance above 70% [11]. Other research studies done in this field have shown that the addition of a conducting polymer, such as polyaniline, into the CNT film enhances electrical conductivity and therefore, improves the performance of the solar cells [25].

**Figure 1:**
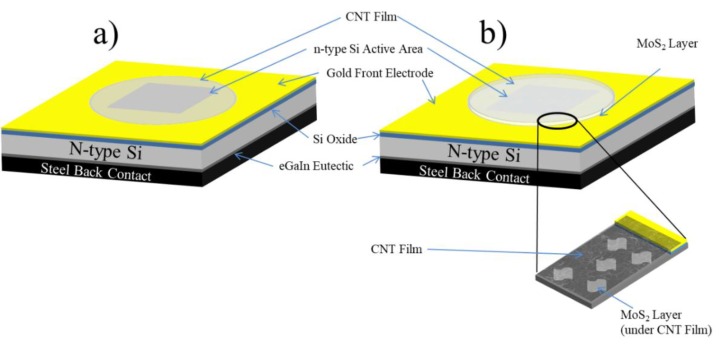
Schematic structure for (**a**) single-wall carbon nanotube (SWCNT)/n-Si solar cells and (**b**) SWCNT/ Molybdenum disulphide (MoS)_2_/n-Si layered solar cells.

There is a need to increase the energy conversion efficiency of photovoltaic systems and given CNTs and MoS_2_ have unique physical properties and it is possible to use simple solution processes for their deposition and application, these materials present an obvious avenue for increasing PCE and achieving high-energy efficiency compared to current solar panels. In this report, the performance of SWCNT/n-Si solar cells will be enhanced with the addition of a MoS_2_ interlayer (see Figure 1) between the CNT layer and the Si substrate (making a SWCNT/MoS_2_/n-Si cell) to help with charge carrier transport.

## 2. Materials and Methods 

### 2.1. Single-Wall Carbon Nanotube Dispersion

A single-wall carbon nanotube stock solution was prepared by dispersing 5 mg arc-discharge powder (P3-SWNT, Carbon Solutions Inc., Riverside, CA, USA) in 50 mL of 1% (*v*/*v*) aqueous Triton X-100 (Sigma-Aldrich, Sydney, Australia) by bath sonication (≈50 W_RMS_ (root mean squared Watts), Elmasonic S 30H (Elma Schmidbauer GmbH, Singen, Germany) for 3 × 1 h intervals at room temperature. The resulting SWCNT suspension was centrifuged for 1 h, at 17,500 g (Beckman Coulter Allegra X-22 Centrifuge (Brea, CA, USA)). Then, the upper two thirds of the supernatants were carefully collected and then centrifuged again in the similar manner as previously, with the bottom residue being discarded. The upper two thirds of the supernatants from this second centrifuge cycle were then collected and combined to yield the stock solution. The remaining third of unsuspended carbon was discarded [26].

### 2.2. Molybdenum Disulphide Dispersion

Molybdenum disulphide aqueous dispersion (FlexeGRAPH, Australian National University, Canberra, Australia) was bath sonicated (S 30H, Elmasonic) for three minutes to make a homogeneous suspension. 12.5 mL of the suspension was diluted with 37.5 mL Milli-Q water (Millipore Corporation, Burlington, MA, USA) (in order to keep the concentration of MoS_2_ suspension at 25 *v*/*v*%). The suspension was then centrifuged (Beckman Coulter Allegra X-22 Centrifuge (Brea, CA, USA)) at 500 g for 10 min, whereby afterwards the upper two thirds of the supernatants was collected and then centrifuged again in the same manner as previously, with the bottom residue being discarded. The upper two thirds of the supernatants from this second centrifuge cycle were then collected to yield the stock solution, with the bottom residue being discarded. 

### 2.3. Preparation of Si Wafer-Photolithography

An n-Si wafer doped with phosphorous was rinsed using acetone and dried under a stream of nitrogen. The resistivity of the 525 μm thick wafer was 1–5 Ωm, with 100 nm thermal oxide layer, (ABC GmbH, Munich, Germany). In a clean room (Class 1000), the Au grid structure with an active area of 0.087 cm^2^ was defined by photolithography [27]. By using spin coating at 3000 rpm for 30 s, a positive photoresist (AZ1518, Micro resist technology GmbH, Munich, Germany) was placed on the Si wafer and then softbaked on a hot plate (AREC heating magnetic stirrer from Rowe Scientific, Lonsdale, SA, Australia) at 100 °C for 50 s. The coated wafer was cooled to room temperature before defining the grid patterns using a mask aligner—EVG 610 (EV Group, Braunau am Inn, Austria). The wafer was then immersed in a developer solution—(AZ 726 MIF, AZ Electronic Materials, GmbH, Munich, Germany) for 15 s to develop photoresist. The wafer was then rinsed with water and dried under a stream of nitrogen gas. The Si wafer post-baking process was done on a hot plate with the pattern defined at 115 °C for 50 s. A Quorumtech Q300T-D sputter coater (Quorumtech, East Sussex, UK), equipped with a quartz crystal microbalance, was used to deposit gold and chromium layers (Au/Cr 145/5 nm) on a silicon wafer to form the metal electrode. The substrate was then immersed in acetone for about 90 min followed by a mild rub with a cotton stick in order to dissolve the photoresist. Solar cell substrates were then prepared by cutting pieces of Si sized 1.5 cm^2^. One drop of buffered oxide etch (6:1 of 40% NH_4_F and 49% hydrofluoric acid (HF), Sigma-Aldrich, Saint Louis, MO, USA) was applied on the active area to remove the SiO_2_ layer on the surface (The SiO_2_ layer was considered removed when the surface expelled the aqueous droplet) [27]. 

### 2.4. Device Fabrication

Nanotube films were prepared using vacuum filtration. This was completed by initially mixing an appropriate amount of SWCNT suspension with milli-Q water (Kansas City, MO, USA) to make a solution of 250 mL which when filtered would yield a film with a transmittance of about 80%. The suspension was vacuum filtered with the aid of a water aspirator through two membranes. The filter paper on the bottom (VSWP Millipore, 0.025 μm pore size, nitrocellulose) was patterned with four holes similar to the size of the desired SWCNT films. The top filter paper (HAWP Millipore Burlington, MA, USA, 0.45 μm pore size, mixed cellulose ester (MCE)) remained unpatterned. The difference in the rate of flow through the filter papers causes preferential flow of solution through the top film where the bottom film is patterned. Thus, the CNTs are stacked by the top film in a similar shape as that of the template film. After the solution passed through both films, it was passed through the filtration media two more times to allow enough CNTs to be retained on the film. After this, Milli-Q water was passed through the CNTs again to remove the surfactants. The template used in these experiments produces four identical 0.5 cm^2^ films in each filtration. One film was deposited on a microscope slide for measurement of sheet resistance and optical transmittance, while the others were attached to solar cells substrates for measurement of cell efficiency. 

For film deposition, the films were cut from the MCE membrane and placed (CNT side down) on the substrate. Wetting was done using a small drop of water and the SWCNT/MCE layer sandwiched between a piece of Teflon (on top of MCE paper) and substrate was clamped by two pieces of glass slides. The substrate was then heated at 80 °C for about 15 min and then cooled in darkness for 30 min. The substrates were then washed in acetone three times (30 min each) to dissolve the MCE membrane; second and third washes with stirring facilitate the removal of the MCE membrane. To complete the preparation of cells, the oxide on the reverse side of all Si pieces was manually removed by scratching. A gallium indium eutectic layer (eGaIn, Sigma-Aldrich, Saint Louis, MO, USA) was then painted on the back surface of Si before attaching a piece of stainless steel as the back contact of the device (see Figure 1). 

The cells were then tested 3 times and further subjected to different post-fabrication treatment procedures. First, a drop of 2% HF was applied on the active area to etch off the SiO_2_ formed between the nanotube film and the Si during the attachment step of the films. This was then followed by treating the SWCNT film with two drops of thionyl chloride (SOCl_2_) which was left to evaporate to increase conductivity. Before testing, the residue was washed with ethanol and dried under a stream of nitrogen. In the last step, the devices were again treated with 2% HF in the same manner as previously described which significantly improved performance [26].

### 2.5. Layered SWCNT/ MoS_2_/n-Si Solar Cells

The dispersions of SWCNTs and MoS_2_ were sonicated for 5 min and 1 h respectively. Then, using vacuum filtration, the SWCNT dispersion was filtered to give a constant thickness (250 µL) CNT film. The MoS_2_ film was then formed on the CNT film using different volumes (100–1000 µL) of the MoS_2_ dispersion filtered through the CNT film already in place on the filter paper. The cells were made by turning the filtered films over such that the MoS_2_ layer was in contact with the Si and then dissolving the filter paper.

### 2.6. Solar Cell Characterization

At each stage of preparation, the solar cells produced were tested by applying voltage to the electrodes under a solar simulator in the absence of natural light. An AM 1.5G filter (obtained from Irvine, CA, USA) was used to filter the light. A silicon reference cell (PV Measurements, from the National Institute of Standards and Technology, Gaithersburg, MD, USA) was used to calibrate the irradiance to be 100 mW cm^−2^ on the surface of the sample. A Keithley 2400 SourceMeter (from Newport Corporation, Solon, OH, USA) was used to acquire data that was captured and sent to a computer with LabVIEW 8.2 (National Instruments, Austin, TX, USA).

### 2.7. Film Characterization

A series of (SWCNT, MoS_2_ and SWCNT over MoS_2_) films were fabricated from each sample using the SWCNT and MoS_2_ solutions and deposited on a glass slide (deposited similarly to the previously described film deposition on silicon). Sheet resistance (R_sheet_) measurements were completed on SWCNT, MoS_2_ and SWCNT on MoS_2_ films using a four-point probe linked to Keithlink Film Resistivity Measurement Tool 1.0 (KeithLink Technology, New Taipei City, Taiwan), four readings on each film at various locations were taken and then results were averaged. Optical absorption spectroscopy (UV-Vis spectroscopy, (Cary 60, Agilent, Santa Clara, CA, USA) was used to determine the transmittance of the films as this affects the amount of light passing through the films and subsequently, the amount of energy produced by the cell. 

Scanning Electron Microscopy (SEM) was used to characterize the surface structure of the nanostructures after fabrication. Dispersions were deposited on Si for examination using an FEI Inspect F50 SEM (Hillsboro, OR, USA). 

Atomic Force Microscopy (AFM) was used to investigate the topographical structure of the nanostructures formed. This imaging technique was specifically applied in order to determine the size and structure of SWCNTs, flakes of MoS_2_ and MoS_2_/SWCNT composites. AFM data was acquired under ambient conditions in tapping mode using a Bruker Multimode8 AFM ( Bruker, Santa Barbara, CA, USA) with Nanoscope V controller. The cantilevers used were Silicon (HQNSC15 Mikromasch) with a fundamental resonance frequency of 200–500 kHz and nominal spring constant of 40 N/m. AFM imaging parameters, including set-point, scan rate and feedback gains, were adjusted to optimize image quality and ensure accurate measurement of flake thickness [28,29]. The scanner was calibrated in x, y and z directions using a Si calibration grid (Bruker model number VGRP: 10 µm pitch, 180 nm depth). All analysis of AFM images was performed using Nanoscope analysis software version 1.4 (Bruker, Santa Barbara, CA, USA). 

Raman spectra was acquired with a Witec alpha300R Raman microscope (Witec, Ulm, Germany) at an excitation wavelength of 532 nm using a ×40 objective with a numerical aperture of 0.60. The gratings used for measurements was either the 1800 grooves/mm or the 600 grooves/mm. Integration times for single Raman spectra ranged between 30 s and 60 s for between 2 and 3 accumulations. Confocal Raman images were also acquired with integration times ranging from 5 s to 10 s per pixel, with each pixel being a Raman spectrum. To generate Raman images, the intensity of a given region in the Raman spectrum, corresponding to the material of interest, is plotted against the *X*-*Y* position of the laser during a surface scan.

## 3. Results and Discussion

**Figure 2:**
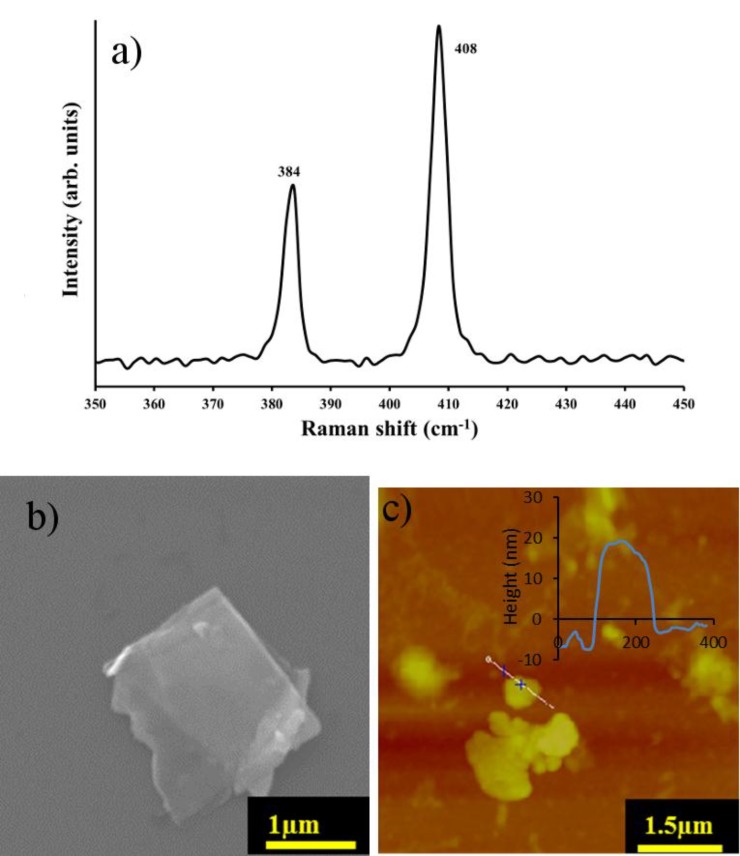
(**a**) Raman spectrum (collected using the 1800 grooves/mm grating); (**b**) Scanning electron microscopy (SEM) image and (**c**) Atomic Force Microscopy (AFM) image of MoS_2_ film deposited on a Si substrate and the corresponding line scan of the MoS_2_-nanosheet film.

The MoS_2_ films used in this work were characterized with representative examples of the results shown in Figure 2.

Raman spectra give a qualitative characterization of MoS_2_ nanosheets. MoS_2_ was deposited on an Si substrate and Raman spectra for the exfoliated MoS_2_ are shown in Figure 2. The expected $E{\;}_{2g}^{1}$ peak at 384 cm^−^^1^ originates from the Mo−S in-plane vibration mode while, the A_1g_ peak is observed near 408 cm^−^^1^ from vibrations of out-of-plane which yields a peak difference between $E{\;}_{2g}^{1}\;$ and A_1g_ of ~25 cm^−^^1^. This is consistent with previously determined values of bulk MoS_2_ [16] where the frequency difference is about 25 cm^−1^ for bulk and 19 cm^−1^ for monolayer [16,30]. Figure 2b displays a typical SEM image of exfoliated MoS_2_ flakes with crystalline straight edges and evidence of partial exfoliation through the semitransparent layers (other representative SEM images are provided in Figure S1). AFM imaging (Figure 2c) shows that the MoS_2_ has a thickness on the order of 25 nm confirming the flakes are many layers thick given the interlayer spacing is ~0.65 nm [16]. The range of lateral size was 100–1000 nm^2^. Raman, SEM and AFM combine to show the exfoliated MoS_2_ is multilayered but remains highly two dimensional.

Similar characterization of the SWCNT film is provided in the supplemental material (Figure S2).

### 3.1. Characterization of Control Solar Cells

**Figure 3:**
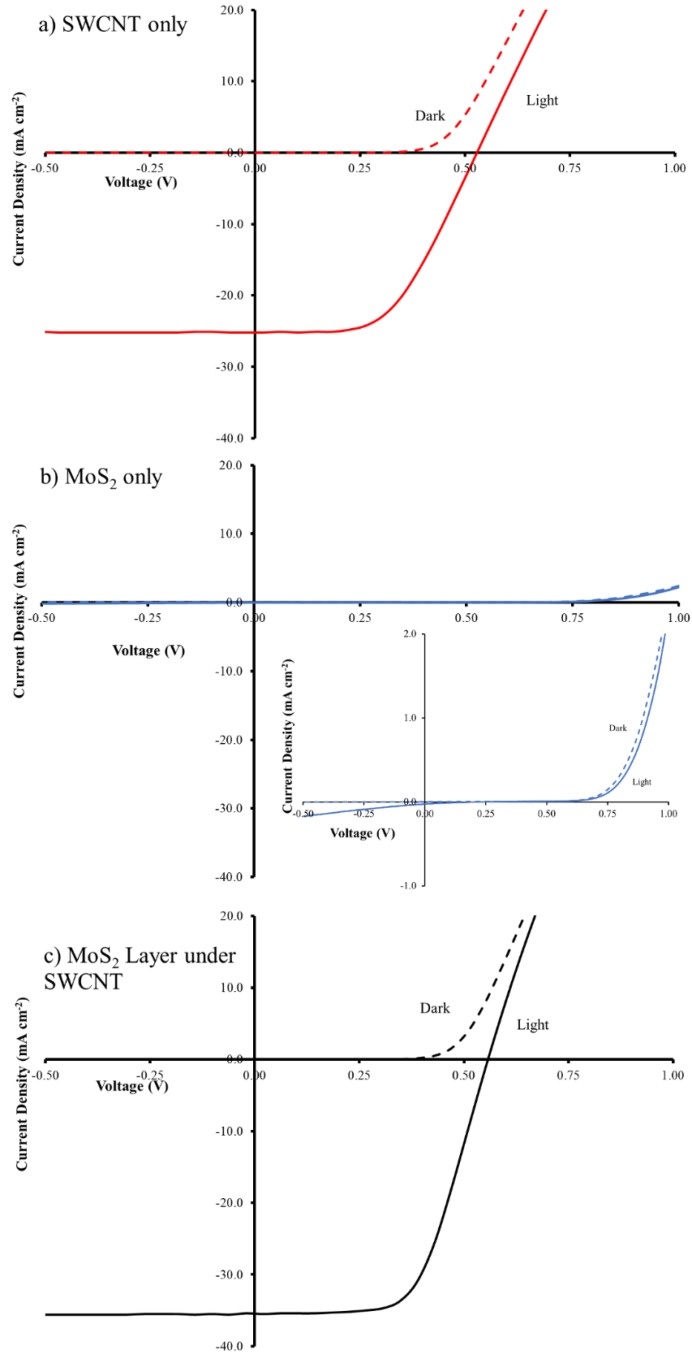
J-V curves of (**a**) SWCNT/n-Si; (**b**) MoS_2_/n-Si and (**c**) SWCNT/MoS_2_/n-Si solar cells. Inset in (**b**) show an expanded view of the J-V curves for MoS_2_/n-Si cells.

In order to investigate the effect of molybdenum disulphide on SWCNT/n-Si solar cells, separate SWCNTs and MoS_2_ aqueous dispersions were prepared and used to fabricate solar cells (SWCNT/n-Si and MoS_2_/n-Si). This experiment was carried out to understand the properties of each material individually for solar cells. Figure 3 illustrates the photocurrent-voltage (J-V) characteristics of the best-performing SWCNT/n-Si and MoS_2_/n-Si solar cells and their detailed photovoltaic performance are summarized in Table 1. It can be seen that SWCNTs only based solar cells exhibit a higher efficiency compared to the MoS_2_ based solar cells. The electrodes made with SWCNTs have a lower sheet resistance than those made with MoS_2_ and hence the SWCNT-based cells exhibit better performance. MoS_2_ can be either an n-type or p-type semiconductor depending on the level of impurities [31]. The MoS_2_ dispersions used in this work are very dilute which lead to very low coverages on the Si substrates. The intimate contact of MoS_2_ and Si in few areas will lead to very few depletion regions with the n-Si being established leading to very low efficiencies.

In order to adjust the thickness of the SWCNT films, the volume of the filtered solutions was varied. Previous work has shown the best CNT film transmittance in CNT/Si solar cells is 75% [24]. A sheet resistance of 531 Ω sq^−1^ is in good agreement with the previously reported value in the literature [11]. On the basis of that, with the current dispersion, 250 µL is an optimal value and its solar cell exhibited an average power conversion efficiency (PCE) of ~6.6% (Table 1). In an effort to improve performance, a very thin layer of MoS_2_ was placed between the n-Si and the SWCNT. Various coverages of MoS_2_ were used to find the optimal conditions. 

### 3.2. Layered SWCNT/MoS_2_/n-Si Solar Cells

**Figure 4:**
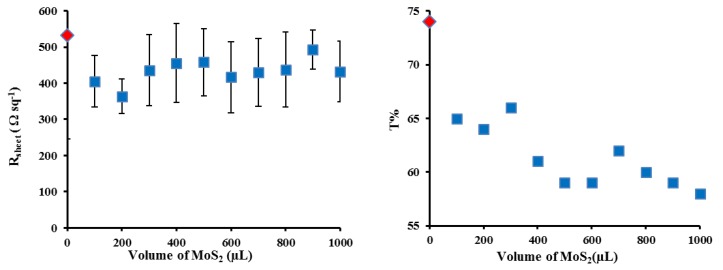
Sheet resistance (**left**) and optical transmittance @550 nm (**right**) of varying layered SWCNTs@ MoS_2_ films thickness after all three chemical treatments.

**Figure 5:**
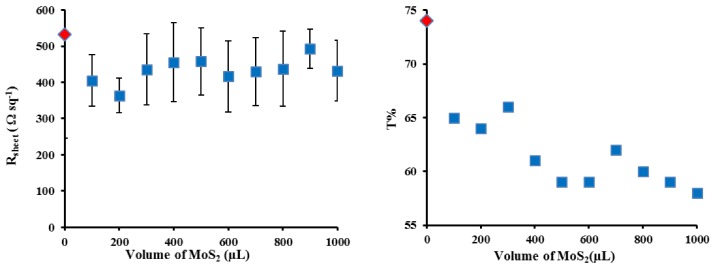

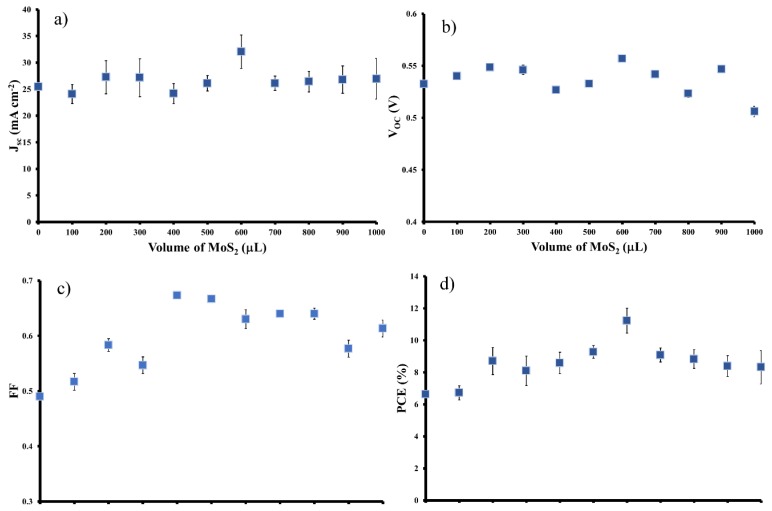
Layered SWCNT/MoS_2_/n-Si solar cells parameters (**a**) J_sc_; (**b**) V_oc_; (**c**) Fill factor (FF) and (**d**) Photoconversion efficiency (PCE) extracted after all three chemical treatments.

The J-V curves for the best cells in Figure 3 show that the addition of the MoS_2_ interlayer leads to significant changes in the short circuit current density (J_sc_) and the fill factor (FF) with a negligible change in open circuit voltage (V_oc_), which leads to a near doubling of the PCE. In order to determine the optimal coverage of the MoS_2_ films, a set of films were prepared by first filtering a constant volume of SWCNT dispersion (250 µL) followed by filtration with different volumes (100–1000 µL) of the MoS_2_ dispersion to give a layered film. Figure 4 shows that the transmittance decreased by ~10% with increasing MoS_2_ volume (from 100 to 1000 µL) as expected as adding more material should increase the film mass and increase the number of scattering elements (leading to more light absorption or scattering). The sheet resistance stayed reasonably constant (Figure 4). Variation in the volume of MoS_2_ dispersion shows a clear maximum in photovoltaic performance when 600 µL of MoS_2_ was used in the filtration to fabricate the layered design. Figure 5 shows that the addition of the MoS_2_ layer leads to small changes in the average J_sc_ while the average V_oc_ is largely unchanged (also see Table 1). The largest changes are observed for the average FF. MoS_2_ has a very long charge carrier diffusion length [12] which should reduce opportunity for charge recombination at junctions such as those between two SWCNTs. The increase in average FF shows that the layers with more MoS_2_ are more effective at extracting the charge carriers and this leads to improve performance. Figure S3 shows that there are no obvious trends in the diode properties (reverse saturation current density (J_sat_) or Ideality) with the values relatively constant for all devices.

The close association of the p-type SWCNTs with the n-type Si substrate is critical for the functioning of these cells as this produces the required depletion region. The addition of an n-type MoS_2_ layer clearly improves the performance (average PCE increase from 6.6% to 11.2%). The concentration of the MoS_2_ is quite low, so at all volumes used the coverage is certainly not complete. This means there are always SWCNT_S_ in contact with the Si to give a working cell. The MoS_2_ flakes are excellent hole conductors and will help improve carrier lifetimes and this will improve performance. The carrier MoS_2_ layer efficiently decreases the recombination loss of carriers which results in an improvement of FF. Once the number of MoS_2_ flakes increases to give reasonably high coverages, the number of nanotubes in close contact with the Si will decrease and performance will decrease again. 

There is a lot of work showing that MoS_2_ density of states shift up and down depending on how it interacts with other materials [32]. This may mean the shift in the band gap of the MoS_2_ provides the opportunity for the MoS_2_ to act as an intermediate facilitating transfer of charge carriers.

## 4. Conclusions

Molybdenum disulphide (MoS_2_) has been successfully used to improve the performance of CNT/Si solar photovoltaics. A number of techniques such as AFM, SEM and Raman were used to characterize the MoS_2_ and CNTs in order to examine their structure, thickness and/or lateral size. The average performance achieved from the CNT/Si-solar cells only was ~6.6% whereas, the highest PCE after the insertion of MoS_2_ layer between SWCNTs and silicon was 11.2%. Addition of the interlayer of molybdenum disulphide led to an improved fill factor which was largely responsible for the improved performance.

## Figures and Tables

**Table 1:** Selected solar cell and diode properties for SWCNTs, MoS_2_ and SWCNT/MoS_2_. Data shown for champion cells (bold typeface) and average properties with standard deviation (regular typeface). Three devices of such type are included in the analysis.

	J_sc_ (mA cm^−2^)	V_oc_ (V)	T%	R_sheet_ (Ω sq^−1^)	FF	Eff (%)
**SWCNT**	**25.17** 25.4 ± 1	**0.529** 0.532 ± 0.009	74	531 ± 74	**0.53** 0.49 ± 0.05	**7.04** 6.6 ± 0.4
**MoS_2_**	**0.025** 0.021 ± 0.004	**0.236** 0.208 ± 0.027	96	1621 ± 236.6	**0.18** 0.2 ± 0.005	**1.06 × 10^−^^3^** 7.99 × 10^-4^ ± 2.67 × 10^-4^
**SWCNT@600 µL MoS_2_**	**35.46** 32.1 ± 3.1	**0.557** 0.557 ± 0.002	59	410 ± 90	**0.61** 0.6 ± 0.01	**12.04** 11.2 ± 0.8

## Supplementary Materials

The following are available online at http://www.mdpi.com/1996-1944/11/4/639/s1, Figure S1: SEM images of various MoS_2_ flakes on Si, Figure S2: (A) Raman spectrum (acquired using the 600 grooves/mm grating and (B) Raman optical image, (C,D) SEM images and (E,F) AFM images of SWCNTs film that were deposited on Si substrates, Figure S3: Diode properties of the devices as a function of the volume of MoS_2_ used.
