# Supplementary material for: Efficiency Improvement Using Molybdenum Disulphide Interlayers in Single-Wall Carbon Nanotube/Silicon Solar Cells
Source: Materials (Basel). 2018 Apr 21;11(4):639. doi: 10.3390/ma11040639 (PMC5951523; doi:10.3390/ma11040639)
Supplement: Supplementary file 1 [file materials-11-00639-s001.pdf]

materials

MDPI

Supplementary

Efficiency Improvement Using Molybdenum Disulphide Interlayers in Single-Wall Carbon Nanotube/Silicon Solar Cells

Shaykha Alzahly [1], LePing Yu [1], Cameron J. Shearer [1,2], Christopher T. Gibson [1] and Joseph G. Shapter [1,3,*]

[1] Flinders Centre for Nanoscale Science and Technology, College of Science and Engineering, Flinders University, Bedford Park, Adelaide, SA 5042, Australia; alza0112@uni.flinders.edu.au (S.A.); yu0252@uni.flinders.edu.au (L.Y.); cameron.shearer@flinders.edu.au (C.J.S.); christopher.gibson@flinders.edu.au (C.T.G.)

[2] Department of Chemistry, The University of Adelaide, Adelaide, SA 5005, Australia

[3] Australian Institute for Bioengineering and Nanotechnology, University of Queensland, St. Lucia, QLD 4072, Australia,

* Correspondence: joe.shapter@flinders.edu.au; Tel.: +61-07-3343-1165

Received: 27 March 2018; Accepted: 12 April 2018; Published: 21 April 2018

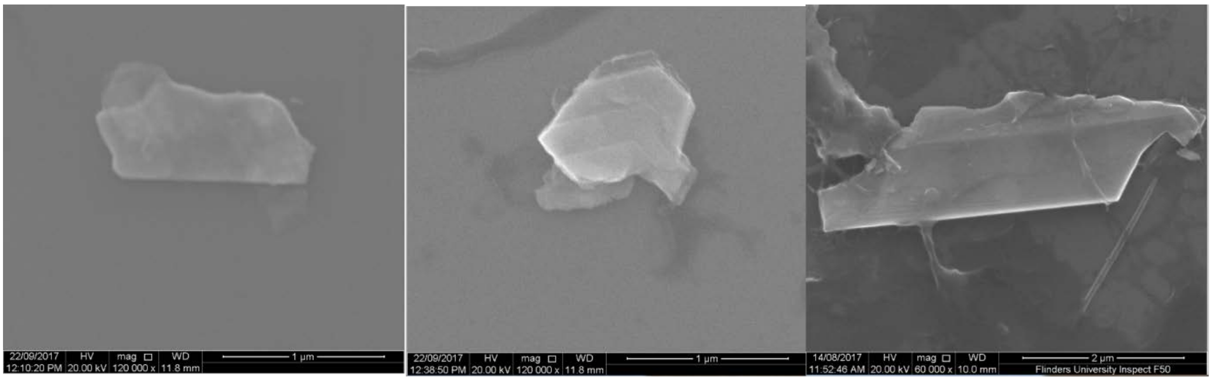

Figure S1. SEM images of various MoS_2 flakes on Si.

Single Walled Carbon Nanotube (SWCNTs)

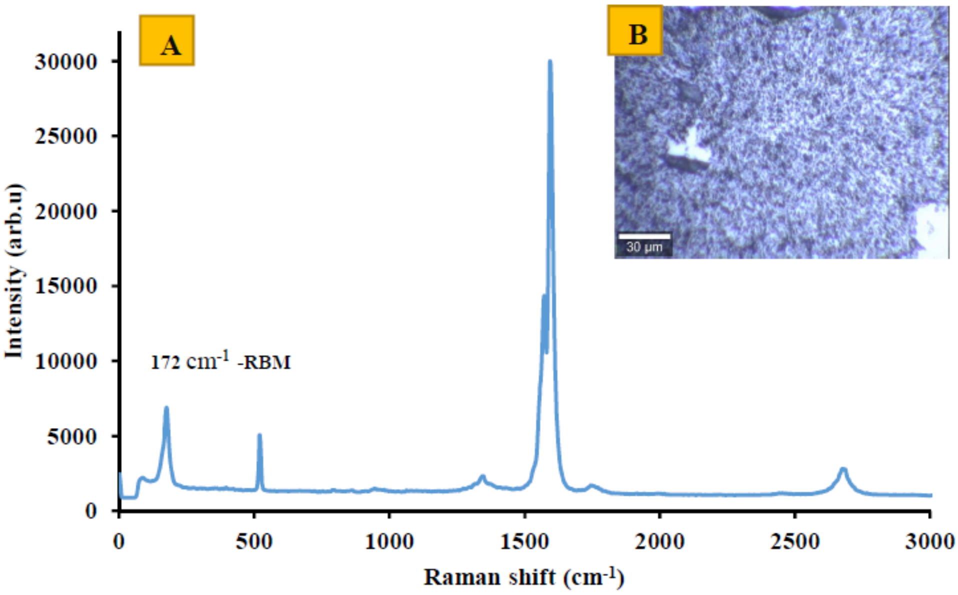

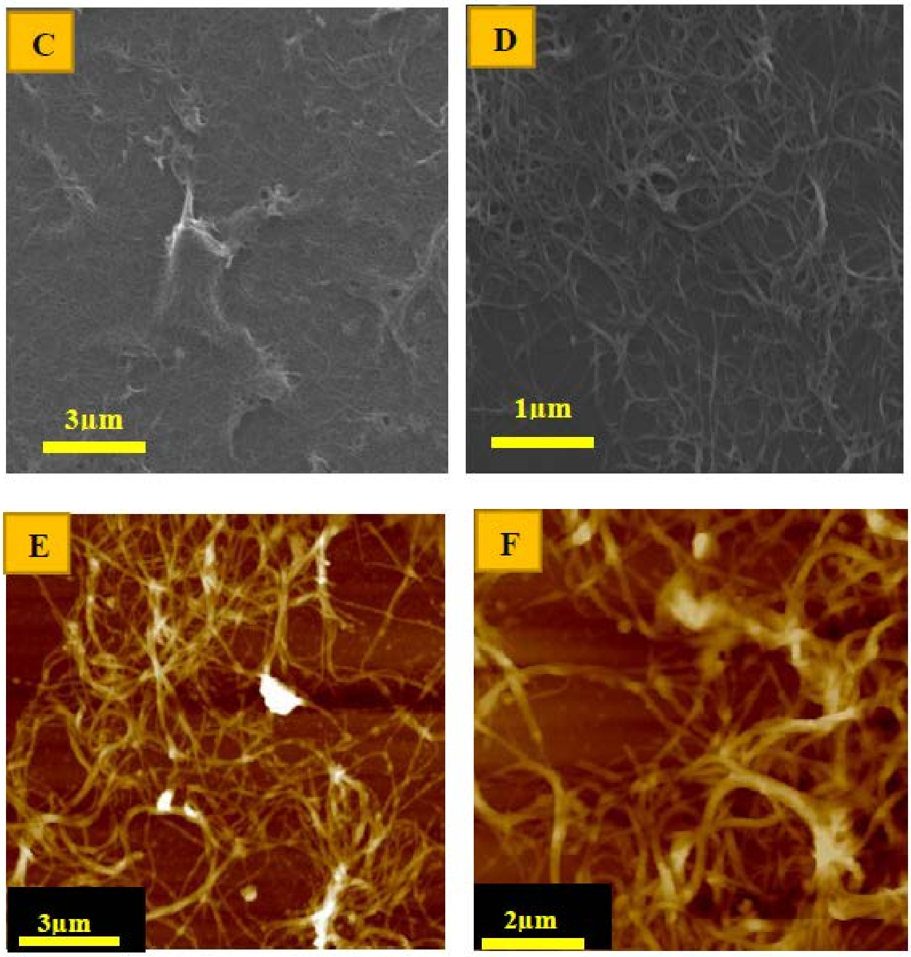

Figure S2. (**A**) Raman spectrum (acquired using the 600 grooves/mm grating and (**B**) Raman optical image, (**C**,**D**) SEM images and (**E**,**F**) AFM images of SWCNTs film that were deposited on Si substrates.

Figure S2A,B is example Raman spectrum and optical images for SWCNTs on a Si substrate. The peaks monitored in the spectrum are those expected for SWCNTs [1]. The peak at 512 cm^{-1} is from the silicon substrate. The Raman spectra proved the presence of SWCNTs on the substrate showing the radial breathing mode (RBM). The location of the RBM (ω_{RBM}) provides information about SWCNTs diameter by using the equation

$$\text{RBM shift (cm}^{-1}) = A/d_t + B^1 \quad (1)$$

Where d_t is diameter of the SWCNT and A B are constants which depend on the SWCNTs environment, have previously been measured and the value of A, B are 234 and 10 cm^{-1} respectively [2]. This expression is used with the RBM peak position and gave a calculated diameter of 1.4 nm; which is in agreement with supplied diameters of the manufacturer. Another common calculation of Raman's SWCNTs is the intensity ratio of the D and G- bands (D/G ratio). The (D/G ratio) is often used to calculate the disorder in CNTs samples and the value is ~0.08.

SWCNT films were deposited on Si substrates and then the films were imaged by scanning electron microscopy (SEM) and atomic force microscopy (AFM) in order to determine the morphology of the films. Figure S2C–F) illustrates SEM and AFM images, respectively.

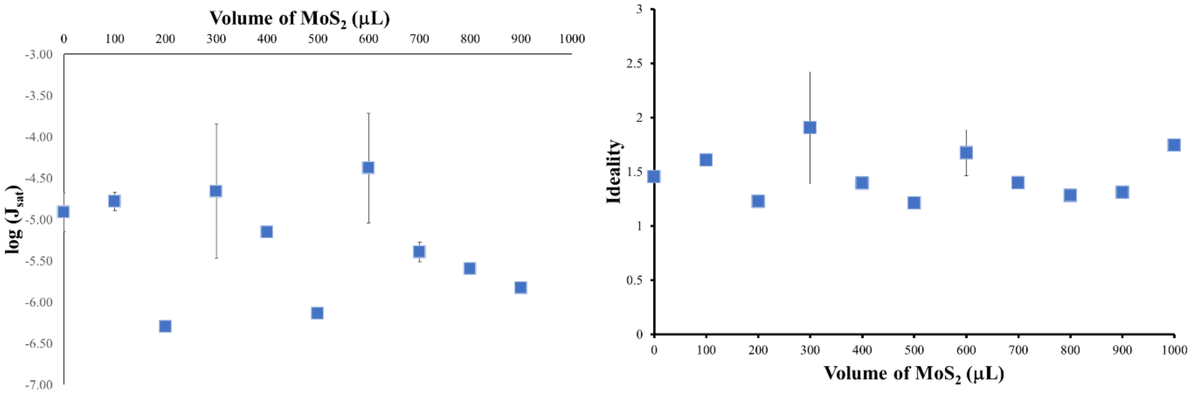

Figure S3. Diode properties of the devices as a function of the volume of MoS_2 used.

Reference

1. Villalpando-Paez, F.; Moura, L.G.; Fantini, C.; Muramatsu, H.; Hayashi, T.; Kim, Y.A.; Endo, M.; Terrones, M.; Pimenta, M.A.; Dresselhaus, M.S. Tunable Raman spectroscopy study of CVD and peapod-derived bundled and individual double-wall carbon nanotubes. *Phys. Rev. B* **2010**, *82*, 155416.
2. Pozegic, T.R.; Anguita, J.V.; Hamerton, I.; Jayawardena, K.D.G.I.; Chen, J.-S.; Stolojan, V.; Ballocchi, P.; Walsh, R.; Silva, S.R.P. Multi-Functional Carbon Fibre Composites using Carbon Nanotubes as an Alternative to Polymer Sizing. *Sci. Rep.* **2016**, *6*, 37334.

© 2018 by the authors. Submitted for possible open access publication under the terms and conditions of the Creative Commons Attribution (CC BY) license (http://creativecommons.org/licenses/by/4.0/).
